# Design of experiments‐guided membrane capture facilitates integration of continuous virus inactivation using Phi6 as a surrogate virus in an intensified downstream process

**DOI:** 10.1002/btpr.88504

**Published:** 2026-03-25

**Authors:** Mario Grünberg, Lisa Lipski, Gabriel Fisicaro, Thomas Duvignau, Karolina Meyer‐Heinrichs, Diana Carmen Mocsy, Thomas‐Josef Filz, Bastian Quaas, Alexandra Stützer

**Affiliations:** ^1^ Department of Separation Technologies Sartorius Goettingen Germany; ^2^ Process Development Pilot and Processing LFB Biomanufacturing Ales France

**Keywords:** affinity chromatography, bacteriophage phi6, continuous virus inactivation, design of experiments, integrated continuous bioprocessing, membrane chromatography, process intensification

## Abstract

The growing demand for cost‐efficient and flexible biomanufacturing has increased interest in process‐intensified downstream platforms. This study evaluates an intensified monoclonal antibody (mAb) purification sequence, where two unit operations traditionally performed in batch mode—Protein A capture and low pH virus inactivation (VI)—are redesigned to enhance productivity and minimize resource usage. Rapid‐cycling Protein A membrane chromatography was optimized using a design‐of‐experiments approach to address inherent membrane challenges such as buffer consumption. Wash step volumes were systematically reduced without compromising host cell protein or host cell DNA clearance, yielding a 70% reduction in total wash buffer consumption. At manufacturing scale, membrane adsorbers achieved critical quality attributes comparable to a benchmark resin, while increasing productivity ~20‐fold and lowering capture‐step costs. Protein A eluates were processed in a novel continuous virus inactivation (cVI) system using bacteriophage Phi6 as a surrogate for enveloped viruses. At residence times of 35 and 70 min, the cVI system achieved a ≥5‐log reduction, equaling conventional batch performance without compromising mAb quality. The study demonstrates that membrane‐based Protein A capture and continuous VI can be seamlessly integrated into an intensified DSP framework. This approach effectively maintains product quality while significantly reducing buffer usage and cost, thus supporting modular intensification strategies for clinical‐scale mAb manufacturing.

## 1 INTRODUCTION

Biopharmaceuticals are becoming increasingly important in disease treatment, with monoclonal antibodies (mAbs) continuing to dominate approvals, representing 53.5% of all biopharmaceuticals.^1^, ^2^ From over 1000 candidates in clinical phase 1, fewer than one‐fifth typically progress to later stages, incurring high costs without generating revenue.^3^ Patent expirations enable biosimilars and biobetters, driving cost‐effective therapies and decentralizing production, though pricing pressure persists.^4^ Intensified downstream processes and automation reduce cleanroom footprint, buffer consumption, and labor, improving efficiency and economic viability.^5^, ^6^, ^7^

**FIGURE 1:**
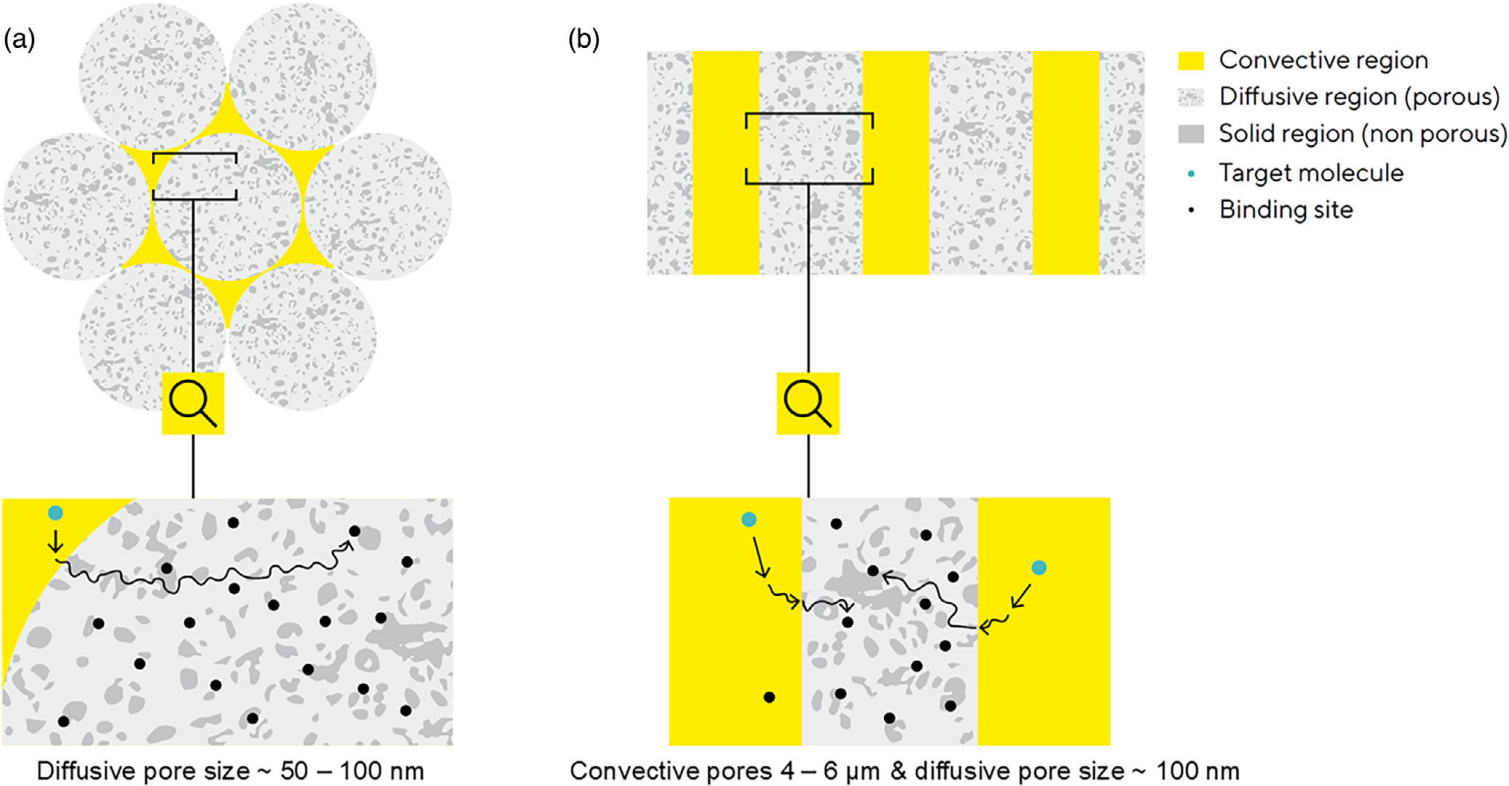
Comparison of chromatographic materials regarding main mass transport mechanism—(a) diffusive mass transport in agarose‐based chromatography resins, (b) agarose‐based membranes (Sartobind® Rapid A) combining diffusive and convective mass transport.

Despite these benefits, the adoption of intensified processing technology within the industry remains slow. Protein A capture serves as a prime example: while multi‐column chromatography and membrane‐based matrices offer resin savings and facilitate scalability, there is hesitancy due to perceived complexity.^8^, ^9^ Membrane protein A adsorbers, such as Sartobind® Rapid A, eliminate the need for column packing and reduce fouling propensity and footprint. Due to its structure, membrane chromatography enables short residence times of 10–30 s versus 3–6 min for resins, allowing rapid product elution and immediate downstream processing (Figure 1).

One of the challenges associated with membrane adsorbers is their elevated buffer consumption. Hydrodynamic effects such as dispersion and back‐mixing contribute to increased buffer usage compared to chromatographic resins.^10^, ^11^ The ratio of hold‐up volumes of common chromatography systems relative to membrane volume is often undesirably high, unlike traditional columns where this issue can be neglected.^12^, ^13^, ^14^ To address these technical limitations, a systematic performance evaluation of MA should be conducted alongside classical resin chromatography. This approach can identify areas where MA properties might surpass resins, leading to buffer reduction and facilitating the broader adoption of intensified membrane chromatography.

Design of experiments (DOE) is a systematic data analysis tool that optimizes performance, improves quality, and reduces variability by efficiently identifying key variables and their interactions. In contrast to a one‐factor‐at‐a‐time approach, DOE facilitates the simultaneous evaluation of several factors, such as the effects of buffer volumes, conductivity, and pH on the removal of host‐cell impurities or yield, while minimizing the number of experiments and ensuring statistical significance.

In addition to optimizing Protein A capture using MA, integrating subsequent unit operations like virus inactivation (VI) is pivotal in advancing process intensification. This integration represents the first level of process intensification, enhancing workflow efficiency and reducing process time. Traditionally virus inactivation is performed in batch‐mode within stirred tanks, but transitioning to continuous implementation can boost efficiency. Despite the availability of semi‐batch technologies, their adoption has been hindered by limited reliable data on continuous or semi‐continuous VI in commercial mAb production. Viral clearance studies typically demand large quantities of viruses, resulting in high costs and increased laboratory safety requirements. For practical reasons virus surrogates like the bacteriophage Phi6 can be utilized as a model for enveloped viruses. As outlined in Table 1, these surrogates are suitable for proof‐of‐concept experiments to assess the general functionality of newly developed biopharmaceutical equipment for low pH cVI. However, it is essential to complement these studies with enveloped model viruses recommended by ICH Q5A(R2),^23^ such as PRV or X‐MuLV, for thorough validation.

**TABLE 1:** Comparison of physicochemical properties of the phage Phi6 with enveloped model viruses PRV and X‐MuLV, and their impact on pH stability and comparability.

Property	Impact on acid stability	PRV and X‐MuLV	Phi6	References
Structure	High	Genome enclosed in protein shell	Genome enclosed in protein shell	15, 16, 17, 18
Genome	Medium	Virally encoded	Virally encoded	15, 17
Envelope	High	Lipid bilayer	Lipid bilayer	15, 16, 19, 20
Envelope thickness	High	~ 4–6 nm	~ 4–6 nm	^21^
Glycosylation	Indirect	Yes	Limited|None	21, 22

This study demonstrates that a membrane‐based capture step is competitive with a state‐of‐the‐art Protein A resin in terms of CQA, CCP, and buffer usage. To illustrate the comparability to conventional batch low pH virus inactivation methods, product eluates were processed in a novel cVI system using bacteriophage Phi6 as a surrogate for enveloped viruses.

## 2 MATERIALS AND METHODS

### 2.1 Cell culture production and clarification

The recombinant human monoclonal antibody (LFB Biomanufacturing, France, proprietary IgG1, isoelectric point 8.5) was expressed in Chinese hamster ovary cells (CHO‐DG44). Cultivations were carried out in 2000 L Biostat® STR bioreactors (Sartorius, Germany) operated in fed‐batch mode for 14 days. Cell culture clarification was performed in two depth filtration steps: a 4.4 m^2^ D0HC cassette followed by a 2.2 m^2^ X0HC cassette (Merck Millipore, Germany). A final sterilizing‐grade filtration was then conducted using a 20 inch Opticap filter (Merck Millipore, Germany). 440 L of harvested cell culture fluid (HCCF) with a titer of 5.50 g/L was utilized for chromatographic purification. The HCCF was stored at 5 ± 3°C to simulate a worst‐case scenario for maximum hold time. Post‐capture, the antibody eluates were frozen at −20°C in 10 L bags since the cVI experiments could not be conducted immediately. Each bag underwent one freeze–thaw cycle.

### 2.2 Protein A chromatography

Protein A MA utilized were Sartobind® Rapid A Nano (1 MV = 1.2 mL) for screening and process development, and Sartobind® Rapid A Cassettes (1 MV = 0.8 L) for manufacturing scale were obtained from Sartorius, Germany. HiScreen™ MabSelect SuRe™ LX columns (1 CV = 4.7 mL) were obtained from Cytiva, Sweden. Benchtop scale IgG capture was conducted on an ÄKTA Avant 150 (Cytiva, Sweden). For scale‐up experiments, two MA cassettes (1.6 L MV) were utilized with the Resolute® Flowdrive SU (Sartorius, UK), a chromatography system with a single‐use flowpath. A column−/membrane guard filter (Sartopore® 2 XLG, Sartorius, Germany) was implemented to prevent fouling and clogging of the stationary phase.

Mass loading was standardized to 34.5 g mAb per liter of membrane or resin. The chromatography recipes were based on the manufacturer's instructions of adsorbers and resins (Table 2). The standard chromatography recipe steps and buffer compositions reflect conventional protein A capture using resins (provided by LFB Biomanufacturing, Ales, France). For the DOE‐optimized recipes, buffer compositions and residence times remained the same, but block volumes for wash steps were based on DOE results. Residence times for the scale‐up experiments (MA, 1.6 L MV) were partially extended due to the pump capacity limitations of the chromatography system.

**TABLE 2:** Process steps of standard and DOE‐optimized recipes with block volumes (in MV or CV) and residence times (in min).

Step	Standard recipe		DOE‐optimized recipe	
	MA	Resin	MA	MA
	1.2 mL MV	4.7 mL CV	1.2 mL MV	1.6 L MV
Equilibration^a^	5 @ 0.1 min	10 @ 4 min	5 @ 0.1 min	5 @ 0.16 min
Load (34.5 g/L)	@ 0.2 min	@ 5 min	@ 0.2 min	@ 0.2 min
Wash1^a^	10 @ 0.1 min	5 @ 4 min	‐	‐
Wash2^b^	10 @ 0.1 min	5 @ 4 min	10 @ 0.1 min	10 @ 0.16 min
Wash3^a^	10 @ 0.1 min	4 @ 4 min	3 @ 0.1 min	3 @ 0.1 min
Elution^c^	15 @ 0.2 min^d^	5 @ 5 min	10 @ 0.2 min^d^	10 @ 0.2 min^e^
Strip^f^	10 @ 0.1 min	2 @ 4 min	‐	‐
CIP^g^, ^h^	4 @ 0.2 min	4 @ 4 min	3.8 @ 0.2 min	3.8 @ 0.2 min
Re‐Eq.^a^, ^h^	5 @ 0.1 min	5 @ 4 min	5 @ 0.1 min	5 @ 0.16 min

*Note*: Recipes for different MA and resin volumes are indicated.

^a^ Equilibration, Wash1, Wash3, Re‐Eq.: 25 mM TRIS, 5 mM EDTA, 25 mM NaCl, pH 7.1 ± 0.1.

^b^ Wash2: 25 mM Tris, 5 mM EDTA, 1.2 M NaCl, pH 7.1 ± 0.1.

^c^ Elution: 50 mM acetic acid, pH 3.5 ± 0.1.

^d^ Peak‐cutting from 50 to 50 mAU/mm.

^e^ Peak‐cutting from 40 to 40 mAU/mm.

^f^ Strip: 0.1 M Acetic Acid, 1 M NaCl, pH 3.0 ± 0.1.

^g^ CIP: 0.2 M NaOH.

^h^ Block ends when target pH of the associated buffer is reached.

### 2.3 Low pH virus inactivation with bacteriophages Phi6

Continuous low pH virus inactivation with Phi6 as surrogate for enveloped viruses was carried out with the cVI system, Pionic® Spin (Sartorius, Germany). The bacteriophage Phi6 was produced in its host strain, *Pseudomonas syringae*, both sourced from the DSMZ strain collection. Acidification was carried out using 30% (v/v) Acetic Acid to achieve a pH set point of pH 3.6 ± 0.1. Neutralization (target: pH 5.0 ± 0.1) was achieved by addition of 1 M Tris–HCl, pH 9. Two flow rates, 12 L/h and 6 L/h, were chosen, corresponding to residence times of 35 and 70 min, respectively, within the incubator chamber.

The flow kit of Incubator 7.0 was customized to enable precise sampling and controlled spiking of Phi6. Six dedicated sampling ports were integrated into the incubator flowkit: one before the incubator inlet, four between the subplates of the incubator, and one at the outlet. A peristaltic pump was installed upstream of the incubator via a T‐connector to enable the continuous spiking of the phage stock solution to achieve a final concentration of 5 × 10^7^ pfu/mL, while a static inline mixer was incorporated into the flow path to ensure homogeneous mixing with the MA eluate. Samples were collected at the designated ports using a back‐to‐front approach to avoid disturbing the fluid dynamics in the incubator, once at least 1.5 chamber volumes of acidified solution had passed through and steady state, indicated by stable pH and flow rates, was achieved.

For comparison, batch virus inactivation was conducted by manually adjusting the pH of the Protein A eluate to pH 3.6 ± 0.1 in a beaker with gentle stirring to prevent vortex formation, followed by addition of bacteriophage Phi6 to a final concentration of 5 × 10^7^ pfu/mL, with samples collected at designated time points as reference for the cVI.

All samples from cVI and batch VI were immediately neutralized with 1 M Tris–HCl pH 9 and analyzed for phage titer and protein aggregation. Plaque assays were applied to quantify phage titers following low pH inactivation. Log reduction values (LRV) were calculated with all results reported to one decimal place.

$$\mathrm{LRV}=\frac{c_{\text{intitial}}}{c_{\text{final}}}$$

If no plaques were observed, a detection (*p* = 0.05) limit of 20 pfu/mL was assumed for a tested sample volume of 150 μL.

$$c_{\text{Phages}}=\ln\frac{p}{-V}=\ln\left(\frac{0.05}{-0.15}\right)=19.97\frac{\mathrm{pfu}}{\mathrm{mL}}\sim 20\frac{\mathrm{pfu}}{\mathrm{mL}}$$

Therefore, based on statistical considerations, a value of 20 pfu/mL is assumed in the evaluation if no countable plaques are present.

Further detailed parameters regarding phage production and inactivation are summarized in the Supporting Information.

### 2.4 Quantification of mAb titer and monomer

MAb titer quantification and high molecular weight/low molecular weight species (HMW/LMW) were analyzed by HPLC size exclusion on an UHPLC system (Vanquish™ Duo, Thermo Fisher Scientific, USA) equipped with a 4.6 mm × 30 cm TSKgel UP‐SW3000 column (Tosoh, Japan). Data analysis was performed using Chromeleon Software (Thermo Fisher Scientific, USA). Monomer levels were determined as a ratio of peak areas of the early‐eluting aggregate peak(s) and late‐eluting fragment peak(s). Additional experimental details pertaining HPLC analysis are provided in the Supporting Information.

### 2.5 Host cell protein, hcDNA and leached protein a measurements

The quantification of host cell proteins (HCP) was performed using a commercial HCP‐ELISA Kit 3G F‐550‐1 (Cygnus Technologies, USA). Host cell DNA (hcDNA) was quantified using the PicoGreen assay P11496 (Thermo Fisher Scientific, USA). The LRV of host cell‐related impurities was determined as the common logarithm of the ratio of impurity concentration [ppm] in the feed (HCCF) and in the elution fraction (product).

$$\mathrm{LRV}=\log10\;\frac{{\mathrm{ppm}}_{\text{HCCF}}}{{\mathrm{ppm}}_{\text{Elution}}}$$

The concentration of leachable Protein A was quantified by a commercial ELISA kit F600 (Cygnus Technologies, USA). For samples obtained from the Sartobind® Rapid A MA, the standard curve was prepared using the lyophilized Protein A ligand (Sartorius, Germany). For resin derived samples, the Protein A standard provided by the kit was utilized. All ELISAs were performed according to the manufacturer's protocol in conjunction with a plate reader (Tecan Infinite M Nano+, Switzerland).

### 2.6 Design of experiments (DOE)

MODDE® 13 Pro software (Sartorius, Sweden) was used to design a screening study aimed at optimizing wash step volumes of the standard Protein A chromatography recipe using a MA. Wash steps 1–3 of the standard recipe were selected as factors (Table 3). The Strip step was excluded from the recipe. All other steps remained consistent with the standard recipe (Table 2, MA 1.2 mL).

**TABLE 3:** Factor settings for the DOE design.

Factor	Unit	Type	Settings
Wash 1	MV	Quantitative	0–10
Wash 2	MV	Quantitative	3–12
Wash 3	MV	Quantitative	3–10

Impurity removal of HCP and hcDNA, along with mAb recovery, was designated as response variables (Table 4). Target values for impurity removal were derived from analytical results of a standard chromatography recipe utilizing a column (Table 2, Resin 4.7 mL). All experimental runs of the DOE design were conducted using a MA Nano (1.2 mL). The eluates of each run were analyzed for recovery, as well as HCP and hcDNA removal. These results were subsequently input into MODDE® for model generation and optimization.

**TABLE 4:** Response settings for the DOE design.

Response	Unit	Condition	Objective	Min	Target	Max
HCP	LRV	Desired	Target	‐	1.90	‐
hcDNA	LRV	Desired	Target	‐	2.06	‐
Recovery	%	Desired	Maximize	‐	‐	100

### 2.7 Cost modeling

The impact of the capture step in a DSP platform process on the total COGs was examined under consideration of this unit operation performed with an MA versus a resin using the BioSolve v8.3 cost analysis software from Biopharm Services, UK. To achieve this, a standard cost model of a full single use (SU) biomanufacturing facility, known as mAb SU BioPhorum TRM, which is included in the software, was modified according to.^24^

## 3 RESULTS

### 3.1 Comparison of resin versus adsorber and scale‐up

To assess the feasibility of transitioning from a resin‐based mAb‐capture step to a membrane‐based approach, benchtop trials were conducted using Protein A resins and MA to establish reference CQAs. Both consumables (4.7 mL Resin, 1.2 mL MA Nano) were tested with a standard platform capture chromatography recipe, consisting of three wash steps (Wash 1–3) following the capture of IgG1 from HCCF. Wash 1 was carried out with equilibration buffer to remove residual media and impurities, while Wash 2 employed a high‐salt buffer to remove resistant impurities, which are either antibody‐associated or non‐specifically bound to the stationary phase. Wash step 3 used equilibration buffer again to rinse out salt and prevent precipitation in the subsequent CIP step.

The eluates from both consumables were evaluated for their efficiency in removing HCP and hcDNA, as well as their product recovery, monomer content, and levels of leached Protein A ligand. In the standard process design, both consumables showed comparable results across all evaluated parameters. While the resin exhibited slightly better HCP removal (+ 0.1 LRV), the membrane adsorber demonstrated a better LRV for hcDNA (Table 5). Due to the smaller elution volumes and consequently higher antibody concentration in resin eluates, the absolute HCP concentration in the resin eluates is higher compared to the MA (Resin: 26,316 ng/mL; MA: 9588 ng/mL). For both CQA, HCP, and hcDNA, the absolute values were notably lower in the eluates of the MA.

**TABLE 5:** Process parameters and product quality attributes for resin and MA.

	Resin	MA	MA
	4.7 mL CV	1.2 mL MV	1.6 L MV
	Standard	Standard	DOE‐optimized
Mass loading [g/L]	34.5	34.5	34.5
Yield [%]	92.5	87.8	95.4
Monomer [%]	94.1	94.6	94.9
HCP reduction [LRV]	1.9	1.8	1.8
hcDNA reduction [LRV]	2.1	2.3	2.4
Leached ligand [ppm]	20.0	2.5	13.3

*Note*: Eluate results for resin and 1.2 mL MA (homogenized from three cycles) represent the standard chromatography recipe, compared against 1.6 L MA (eluates homogenized from 40 cycles) using the DOE‐optimized recipe.

**FIGURE 2:**
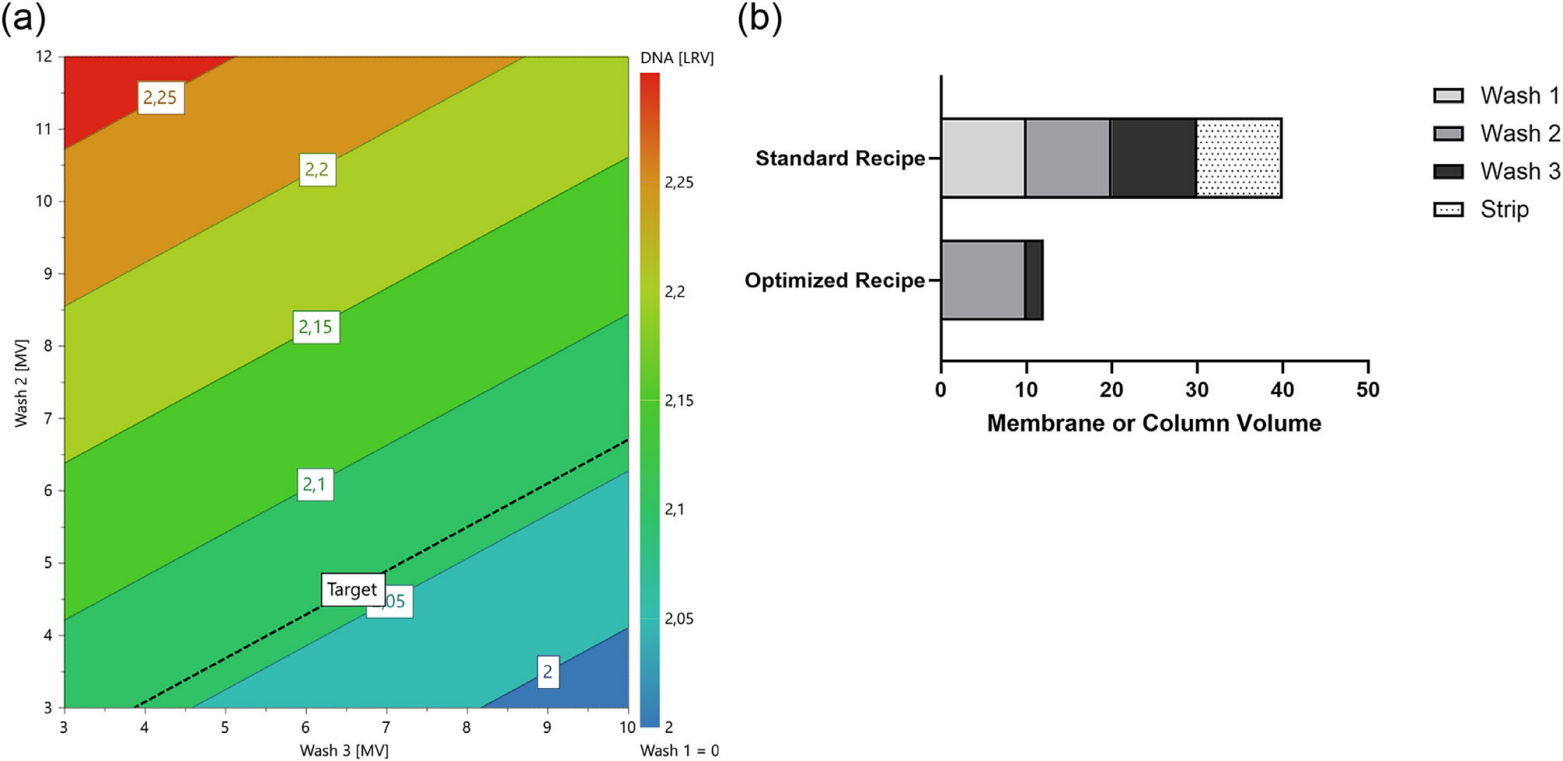
(a) MODDE® Pro 13 contour plot with the two factors of Wash 2 and Wash 3 membrane volumes (MV) according to the model predicted for DNA removal (LRV). Wash 1 was set to 0 MV. (b) Buffer volumes consumed in the wash and strip steps of the standard resin recipe and the DOE‐optimized MA recipe.

The results from the resin reference process were established as the target performance criteria for the DOE using a membrane adsorber. The objective of the DOE was to quantitatively minimize the volume of the three wash steps while maintaining product quality comparable to the resin in terms of yield, HCP, and hcDNA removal. A full factorial experimental design with 2 levels as an interaction model and 3 center points was chosen, resulting in a total of 11 chromatography runs using a small MA (1.2 mL MV). The experimental results showed that the volume of the individual wash steps has no impact on HCP removal (data not shown) but significantly influenced hcDNA reduction. Here, a strong model fit was achieved (R2 = 0.975; Q2 = 0.935; Model validity = 0.671 and Reproducibility = 0.988) indicating that the Wash 1 step can be omitted, the Wash 2 step (high‐salt buffer) should be maximized, and the Wash 3 step can be minimized (Figure 2a). The Strip step of the standard recipe was entirely removed from the DOE design, as a CIP step is sufficient for MA. For the optimized recipe with membrane adsorbers, buffer usage for the wash steps was reduced by 70% compared to the standard recipe, with Wash 1 set to 0 MV, Wash 2 to 10 MV, Wash 3 to 2 MV, and Strip to 0 MV (Figure 2b).

The optimized chromatography recipe was used for a scale‐up with 1.6 L of membrane adsorber cassettes, utilizing the same feed (HCCF) material as used for the benchtop and DOE trials. For the capture step of 400 L of HCCF, two MA cassettes (2 × 0.8 L MV) were deployed over 40 consecutive capture cycles. The residence times varied at 0.20 and 0.16 min, respectively, depending on the chromatography phase. Table 5 presents a comparison of the analytical results from the scale‐up trial with those from the benchtop trials using resin and MA with the standard recipe. As anticipated, the DOE‐based recipe yielded highly comparable results during the scale‐up run, demonstrating the feasibility of transitioning from small‐scale MA (1.2 mL MV) to commercial scale MA (1.6 L MV) operations based on DOE. To accommodate process design considerations, the mass loading was set below the DBC of the tested matrices. Differences in peak cutting and dispersion, arising from system differences, may have contributed to the observed yield discrepancies for the small and large‐scale MA run.

**FIGURE 3:**
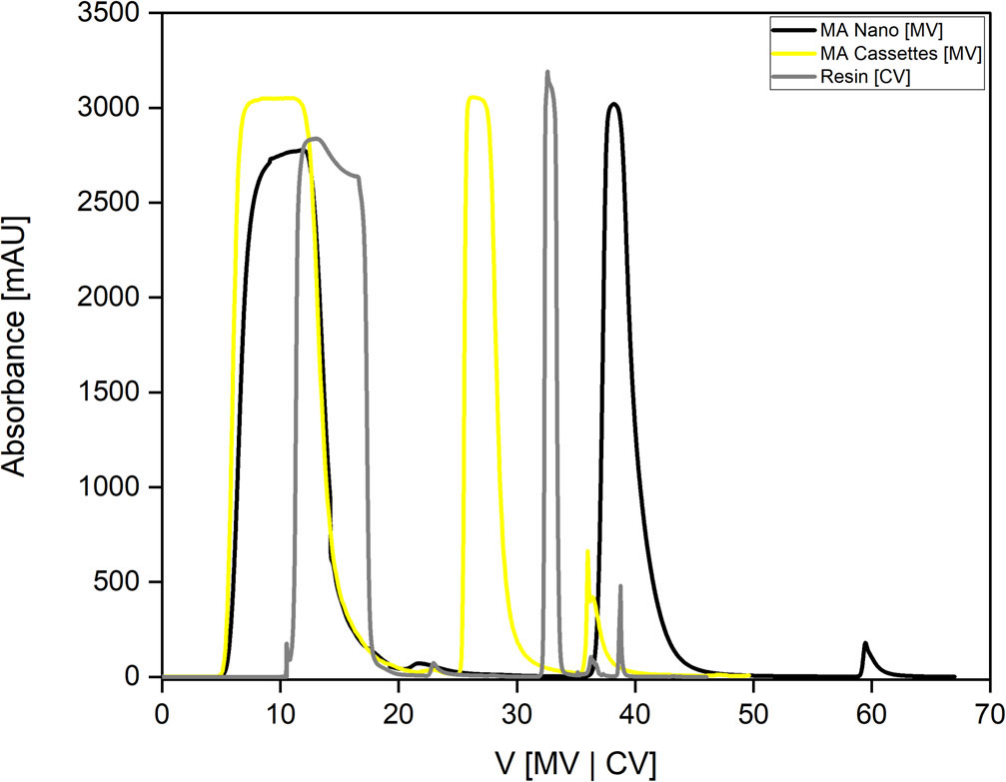
Overlay of UV absorbance spectra at λ = 280 nm of MA 1.2 mL and MA cassettes 1.6 L as well as resin, with one exemplary cycle aligned to the injection point.

The UV curves of the three tested stationary phases show the typical course of a Protein A capture (Figure 3). To facilitate comparison, the volumes were normalized to membrane volumes (MV) for the MA and column volumes (CV) for the resin. Minor peaks were detected in the washing step and more distinct peaks in the CIP step using caustic. The resin exhibited sharper UV signal transitions, whereas the two tested adsorber sizes generated highly congruent UV traces, demonstrating their scalability.

Additional performance indicators were evaluated for the small and large‐scale runs. Table 6 summarizes that the use of MA with the standard recipe enhances productivity about 14‐fold, albeit with a 54% increase in buffer consumption compared to the resin. While the standard recipe's productivity gains were coupled with increased buffer consumption, the optimized chromatography recipe applied to the MA cassettes led to a 35% reduction in buffer consumption per gram of purified mAb compared to the standard adsorber capture step. Additionally, it enhanced productivity 20‐fold compared to the standard resin process. To ensure comparability across the different granularities of chromatographic materials, the amount of purified antibody was normalized to 1 L of stationary phase by extrapolating the resin or membrane volume of the small‐scale trials (resin 4.7 mL, MA 1.2 mL) to 1 liter, or scaling down for the MA cassettes, taking the yield into account.

**TABLE 6:** Product and process parameters for resin and MA.

	Resin	MA	MA
	4.7 mL CV	1.2 mL MV	1.6 L MV
	Standard	Standard	DOE‐optimized
Cycle time [min]	194.6	12.9	9.8
Residence time load [min]	5.0	0.2	0.2
mAb/cycle [g]	0.15	0.04	52.5
mAb/cycle [g/L stationary phase]	31.0	29.8	32.8
Buffer consumption [L/g_mAb_]	1.3	2.0	1.3
Productivity [g/L/h]	9.6	138.8	200.9

*Note*: Results for resin and 1.2 mL represent the standard chromatography recipe, compared against 1.6 L MA using the DOE‐optimized recipe.

Overall, the buffer consumption within the membrane adsorber scale‐up could be reduced while CQAs remained stable or improved. This advancement positions the membrane adsorber on par with resin in terms of buffer efficiency. Additionally, the reduction in cycle times, resulting from fewer and shorter wash steps, significantly increased productivity for the optimized capture recipe.

### 3.2 Surrogate continuous virus inactivation

**FIGURE 4:**
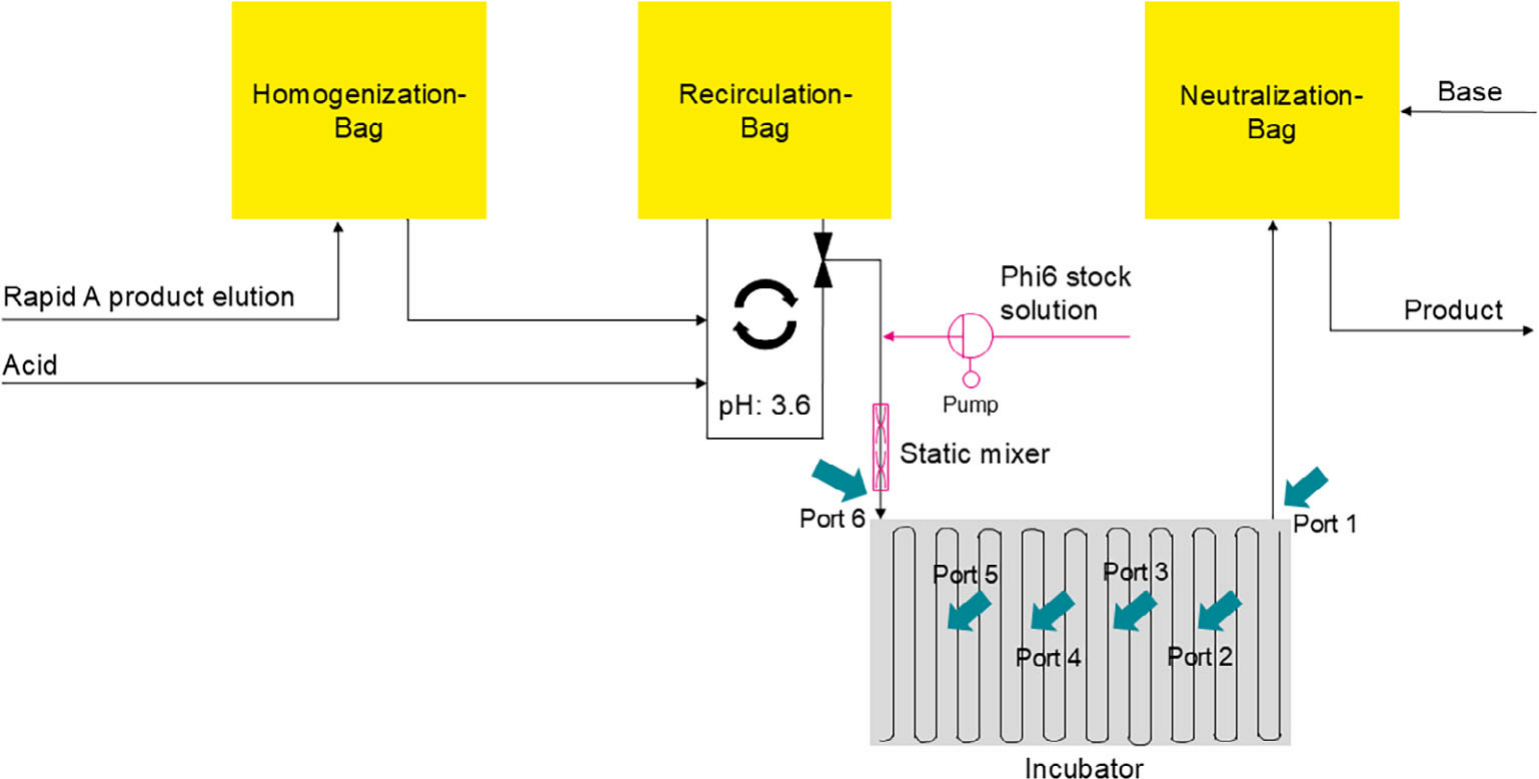
Schematic experimental setup for low pH virus inactivation of Protein A eluates using the Pionic® Spin system and Phi6 bacteriophage. Back‐to‐front sampling was applied (Port 1 to Port 6) to minimize the influence of sampling on the fluid dynamics and residence time.

The Protein A eluates from the MA scale‐up run were pooled and subjected to continuous low pH virus inactivation (cVI) using the Pionic® Spin system. The cVI system continuously titrates acid into incoming Protein A eluates to reach the desired pH within the recirculation loop. The acidified mAb solution then passes through the incubator chamber, followed by neutralization with base in a receiving neutralization bag (Figure 4).

The cVI process was performed at two residence times: 70 and 35 min, corresponding to incubator flow rates of 6 and 12 L/h, respectively. Bacteriophage Phi6 was used as a model virus and continuously injected upstream of the incubator chamber into the acidified solution at 5 × 10^7^ pfu/mL. The serpentine‐shaped incubator promotes the formation of secondary flows (Dean vortices), thereby ensuring a narrow residence time distribution that is essential for reliable virus inactivation. Samples were collected at six ports distributed along the incubator's flow path, corresponding to defined time points post‐phage addition. These samples were analyzed for phage inactivation, with phage clearance calculated as LRV for each time point. In parallel, batch phage inactivation was conducted for total durations of 35 and 70 min. Both the cVI and the batch VI process achieved LRV ≥5 at both residence times, corresponding to a viral clearance of >99.999% (Table 7). Notably, the cVI outperformed batch VI, achieving higher LRV at each residence time.

**TABLE 7:** Viral clearance in LRV achieved with Phi6 in continuous and batch virus low pH inactivation at indicated residence times.

Residence time	Continuous VI	Batch VI
	[LRV]	[LRV]
35 min	6.18	5.68
70 min	5.55	5.06

*Note*: For the cVI process, samples were taken after the incubator (Port 1).

**FIGURE 5:**
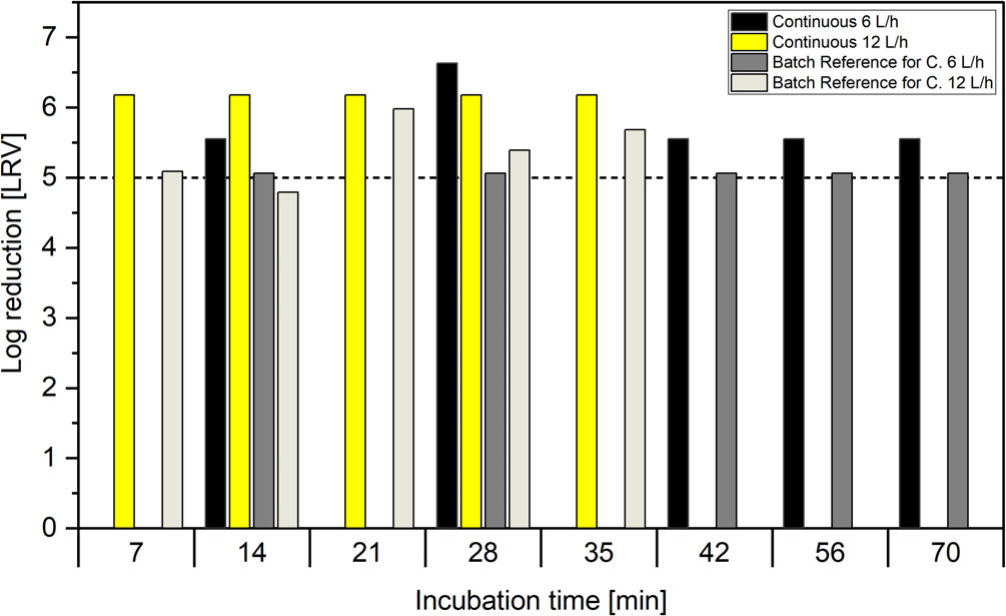
Log‐reduction‐values (LRV) of Phi6 depletion as a function of low pH VI duration under continuous and batch conditions. For cVI, incubation times correspond to specific sampling ports and vary based on flow rates (e.g., port 6 equates to 7 min incubation time at 12 L/h and 14 min at 6 L/h). Batch VI reference processes were sampled at identical time intervals. Threshold of 5 LRV is indicated by a dotted line.

Sampling at different time points post‐phage addition revealed that for both residence times and respective flow rates, a virus clearance of LRV ≥5 was achieved within 28 min or less of exposure to low pH conditions, under both continuous and batch conditions (Figure 5). For the cVI approach, residence times as short as 7 min were sufficient to reach this level of phage clearance. This confirms the rapid and effective inactivation of Phi6 under acidic conditions. Throughout the VI process, HCP and hcDNA levels remained stable, indicating no adverse effects from the inactivation conditions (data not shown).

The results from the cVI system closely matched those from benchtop batch VI trials, demonstrating successful transferability of the phage inactivation performance from batch to continuous processing. Analysis of mAb concentration and purity showed no significant changes in post‐inactivation. Minor increases in HMW species were observed but remained within acceptable limits. LMW species were present from the start and did not increase significantly.

### 3.3 COGs analysis

**FIGURE 6:**
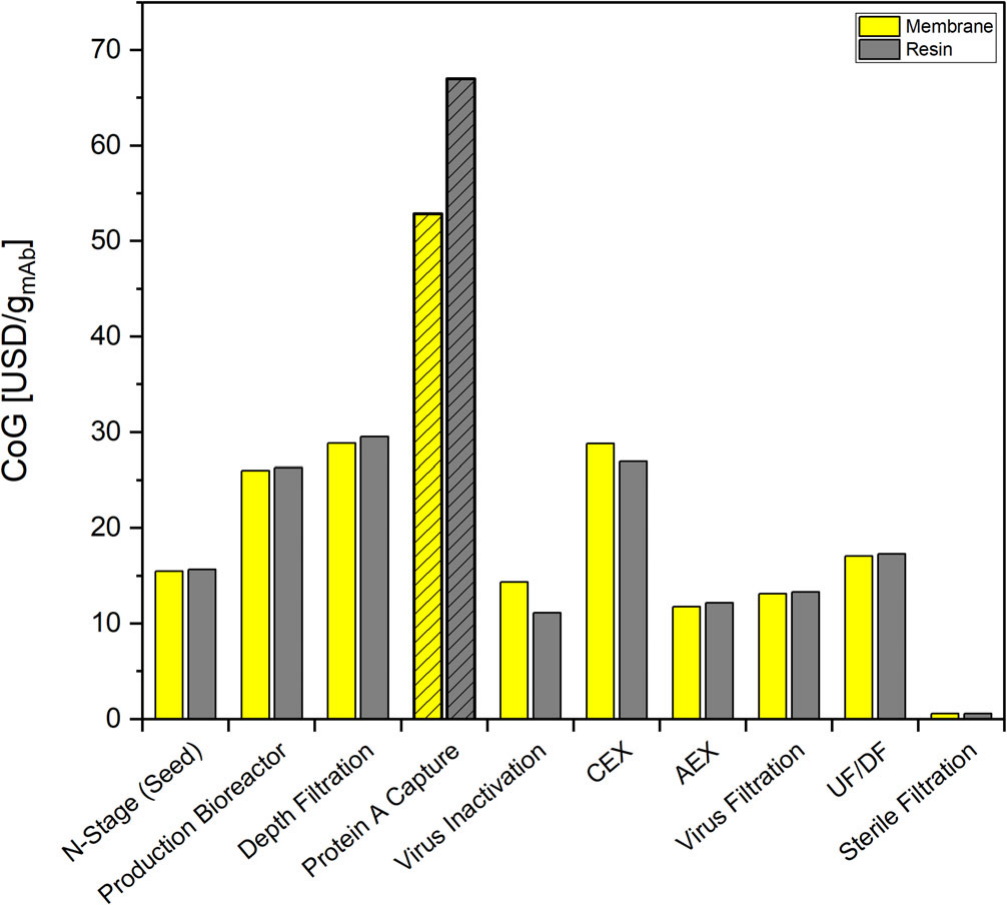
Cost of goods (CoGs) analysis along a mAb process train, assuming SU downstream processing of the output from a 2000 L bioreactor operated for five batches per year to produce (pre‐)clinical material. Highlighted is the effect on the COGs for the Protein A capture step.

Based on the data generated from the experiments with Resin and MA, a comprehensive cost analysis was executed for the individual unit operations of a typical mAb downstream process (Figure 6) using BioSolve v8.3 cost analysis software. The analysis revealed that the impact of an individual unit operation is bidirectional along the process flow, such that changes in its cost affect not only subsequent unit operations but also preceding ones.

The cost modeling assumed identical materials and procedures for all unit operations, either currently in use or planned, with the exception of the capture step. For the resin scenario the following parameters were considered: Resin lifetime of 1 batch comprising 100 cycles; resin footprint (capture) of 5.2 m^2^, which includes skid (2.7 m^2^), column (1.4 m^2^), and receiving area (1.1 m^2^). Buffer tanks were excluded as they are assigned to the Solution Area in BioSolve. For the MA scenario, parameters included: MA lifetime of 1 batch, equating to 102 cycles (calculated for the scenario); MA footprint (capture) of 3.4 m^2^, comprising skid (1.8 m^2^), MA holder (0.5 m^2^), receiving area (1.1 m^2^), with buffer tanks similarly excluded.

Due to the current prototype status of Pionic® Spin and the associated unavailability of pricing information, the manufacturer's batch virus inactivation process continued to be applied to the model. There are marginal differences in the COGs, except for the capture step, the low pH virus inactivation (VI), and to a minor extent for the cation exchange step in bind‐and‐elute mode.

The Protein A step shows the most significant difference under the selected conditions (Resin 67.0 USD/g_mAb_; MA 52.9 USD/g_mAb_). In the context of clinical production, the MA is more cost‐effective because it requires a smaller volume of stationary phase to purify the same amount of material. The model also accounts for the elimination of an empty chromatography column, which is an additional cost item for the resin. Although packing, labor costs for column packing, and column storage are not considered in this cost reduction estimation, they could further increase costs for resin. Consequently, the use of pre‐packed columns has a negligible impact on cost under the specified parameters. The COGs for the capture have been optimized in favor of MAs due to the significant buffer savings, achieved through an optimized capture recipe developed via DOE (eliminated Wash 1 and reduced Wash 3, as described in Figure 2).

The broader elution peak width of the MA (4.9 MV) and its lower product concentration compared to the resin (1.5 CV) necessitate larger volumes for bags, tanks, and buffers for inactivation and neutralization, leading to higher COGs for the VI step. This impacts subsequent unit operations in favor of the resin, as the increased working volume and duration with the MA reduce the productivity of the step. However, for subsequent unit operations, this is almost entirely compensated for by the CEX step, which operates in bind‐and‐elute mode, regardless of the output volume.

In summary, the total costs of producing 55 kg of clinical material annually are 220.0 USD/g_mAb_ for resin and 208.9 USD/g_mAb_ for the MA applying a DOE‐optimized capture step.

## 4 DISCUSSION

### 4.1 Leveraging membrane technology for intensified Protein A capture

In the rapidly evolving landscape of biomanufacturing, process intensification (PI) has shifted from a futuristic concept to a strategic necessity, particularly within clinical manufacturing of mAbs. For early‐phase development, the value of PI lies not just in yield, but in the radical reduction of capital risk and the acceleration of First‐in‐Human timelines. A meaningful entry point into this journey is the intensification of the Protein A capture step using membrane adsorbers. As upstream titers continue to rise, traditional resin‐based chromatography often becomes a restrictive bottleneck, plagued by slow diffusion and large footprint requirements. By adopting convective membrane technologies, manufacturers can transition to rapid‐cycling chromatography, eliminating complex column packing and achieving higher volumetric productivity with a fraction of the chromatographic matrix.

Our data shows that yield and main CQA between resin and MA are highly comparable, yielding almost identical product quality. To maximize this efficiency, we leverage DOE to optimize wash protocols, effectively neutralizing the historically higher buffer consumption associated with membrane‐based systems. While elution pool CQA are similar, MA exhibits stronger hcDNA reduction likely due to its convective mass transfer mechanism, which minimizes non‐specific binding. Conversely, as HCP removal depends on disrupting hydrophobic or ionic antibody and matrix interactions,^25^ clearance is less influenced by convective mass transfer. Consequently, high‐salt wash steps remain essential for effective HCP removal. A distinct mechanical advantage of the membrane structure is its ability to withstand high‐salt washes at elevated flow rates without the pressure‐induced bed compression or channeling common in packed‐bed columns when the binding sites are fully saturated.^26^ By eliminating the need for protective flow‐rate reductions and the intermediate low‐salt transition steps required for resins, MA ensures robust process stability and significantly higher throughput during the critical capture stage. This mechanical resilience was further validated by our DOE results, which confirmed the complete removal of “Wash 1” and the reduction of “Wash 3” (both protective buffer transition steps) without compromising product purity. As demonstrated, the strategic use of DOE facilitates the adoption of new technologies by neutralizing the perceived drawbacks of innovation.^27^

Despite their technical advantages, the large‐scale implementation of MAs in bind‐and‐elute mode for commercial intensified processes remains limited by a lack of comprehensive operational cost data.^28^ This knowledge gap is critical because the Protein A capture step fundamentally defines the lower limit COGs for mAb therapeutics.^29^ Given that Protein A resins typically account for 40%–50% of downstream COGs in traditional batch processing,^30^, ^31^ optimizing this unit operation through tools like DOE is essential. By quantifying the economic benefits of rapid‐cycling MA, manufacturers can better justify the transition from established resin‐based platforms to intensified, membrane‐driven alternatives.

### 4.2 Economic viability of intensified downstream platforms

It is critical to recognize that cost analysis should not be used for generic technology evaluations, as minor variations in process targets or parameters can drastically shift unit operation economics. However, when assessing overall process costs for specific variables, cost analysis becomes a high‐impact tool for identifying primary cost drivers and the dependent influences of successive unit operations.

The scale‐up parameters derived from our intensified process (Table 6) highlight a strong contrast in material utilization. To capture the antibody from a 2000 L bioreactor, approximately 13 L of resin can be substituted by just 1.6 L of membrane. Under these conditions, the resin requires 20 cycles (of a 100‐cycle lifetime) and remains significantly underutilized. In contrast, the membrane achieves 160 cycles, reaching approximately 50% of its proven 300‐cycle lifetime (estimated lifetime: 400 cycles). While the membrane concept targets maximum utilization of the chromatographic material, traditional resin platforms often lead to costly underutilization in clinical settings. The economic tipping point in favor of resin is typically only reached when transitioning to large‐scale commercial manufacturing or high‐volume markets. Conversely, for clinical phases, orphan drugs, or biosimilars with lower demand, the membrane's ability to minimize stationary phase volume makes it the more economic choice.

Each year, hundreds of drug candidates enter clinical phases, typically requiring only a limited number of batches.^32^ The COGs for the batch approach displayed in Figure 5 are below the reported average for (pre‐)clinical material.^33^ Similarly, biosimilars often involve lower production volumes across decentralized, smaller plants to reduce capital investment, further highlighting the relevance of PI.^34^, ^35^ Despite these benefits, the shift from batch to intensified or even continuous processing is often adopted hesitantly; concerns regarding regulatory compliance and the availability of reliable PAT can make the transition time‐consuming and temporarily affect profitability.^36^

### 4.3 De‐risking continuous virus inactivation using bacteriophage Phi6

**FIGURE 7:**
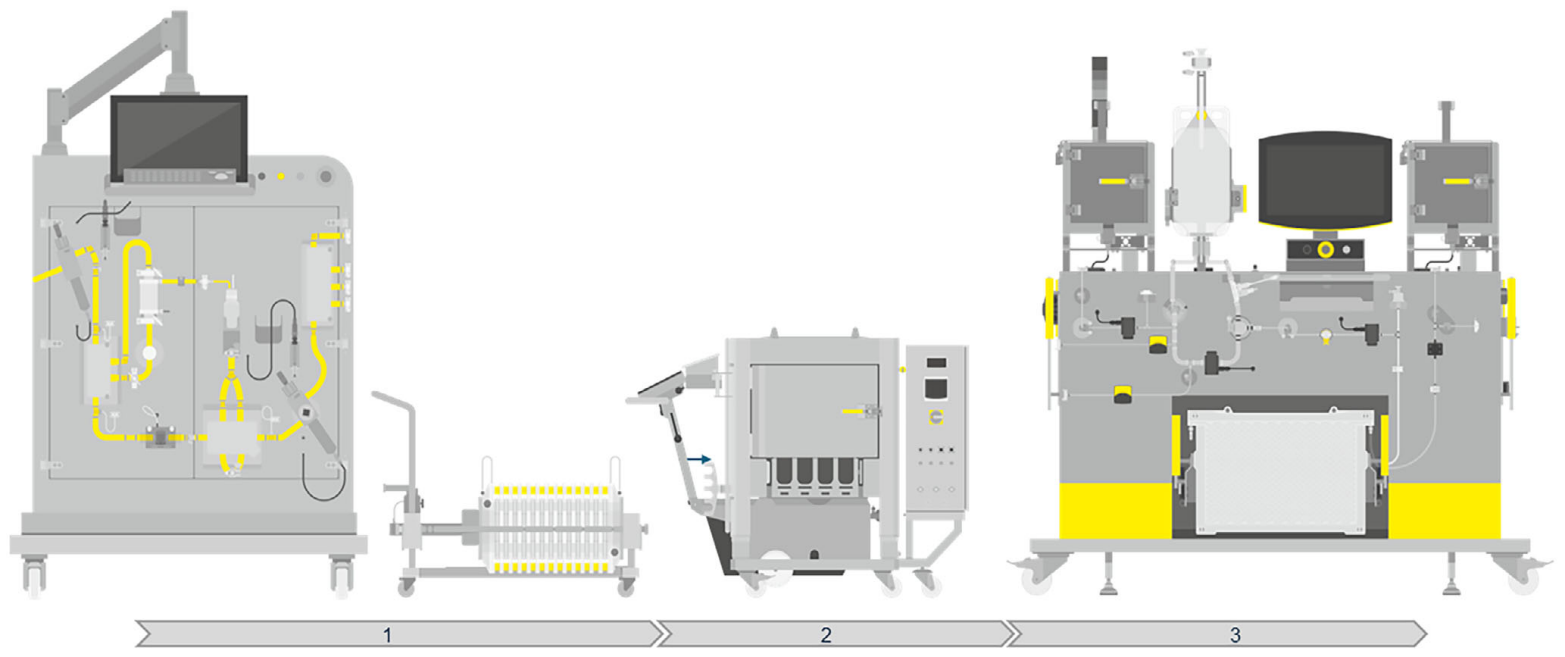
Intensified DSP process with (1) affinity chromatography mAb capture with a single‐use chromatography system and Protein A MA cassettes in a cassette holder, (2) an intermediate surge tank collecting eluates from Protein A chromatography, and (3) a continuous low pH virus inactivation system.

To address these adoption barriers, this proof‐of‐concept study demonstrates a robust technical framework for intensified (Level 1) downstream processing at manufacturing scale. By integrating rapid‐cycling membrane‐based Protein A capture with the Pionic® Spin continuous virus inactivation (cVI) system (Figure 7), we show that increased automation and continuous operation do not compromise viral safety. On the contrary, the low‐pH cVI technology enables tighter process control over critical inactivation parameters, such as pH, mixing, and residence time distribution, providing the high‐resolution data necessary to satisfy evolving regulatory expectations.

A key element of this evaluation was the implementation of bacteriophage Phi6 as a viral surrogate. While regulatory clearance claims eventually require ICH Q5A(R2)‐recommended mammalian viruses like PRV or X‐MuLV,^37^ Phi6 serves as a pragmatic model for early characterization of cVI. By accurately reflecting the physicochemical vulnerabilities of enveloped mammalian viruses at low pH, Phi6 bridges the gap between biological relevance and experimental feasibility. Its use enables large‐scale, process‐oriented studies while bypassing the high costs and restrictive biosafety constraints associated with mammalian‐born viruses.

The bacteriophage Phi6 has been recognized as a suitable surrogate for enveloped viruses, including high‐risk pathogens such as Ebola and SARS‐CoV‐2.^38^, ^39^ Its lipid envelope and structural similarities to mammalian viruses, including a comparable bilayer and envelope thickness,^16^, ^19^, ^20^ make it a valuable model for evaluating inactivation strategies. While Phi6 has been applied in surface decontamination and hygiene studies, its application in pH‐induced inactivation in liquid media remains underexplored. Research indicates that the phage and its nucleocapsid maintain integrity within a pH range of 6.0–9.5.^40^ In droplets, the survival rates of Phi6 depend on the ambient humidity at pH 4.0 and are reduced by 5–6 LRV after 1 h.^41^ While bacteriophage Phi6 lacks the extensive glycosylation of mammalian viruses, which may influence stability under acidic stress,^22^ the results of the present study provide experimental evidence that it can be inactivated effectively and reproducibly in a continuous virus inactivation system. The observed inactivation kinetics, achieving ≥5 LRV at the earliest 7‐min timepoint, show strong alignment with conventional batch VI processes. For comparison, established model viruses such as PRV and X‐MuLV typically reach 4 to 6 LRV within 5–10 min of low‐pH treatment.^42^ Non‐traditional approaches have demonstrated that ≥4.3 LRV for X‐MuLV can be achieved at pH 3.3–3.6 after only 2 min using a static mixer,^43^ and that no residual infectivity of PRV or X‐MuLV could be detected following 15 min of exposure at pH 3.7 in a packed‐bed continuous virus inactivation reactor.^44^ These findings suggest that Phi6 serves as a reliable surrogate for enveloped viruses for characterization of continuous low‐pH inactivation, while acknowledging that differences in envelope composition between bacteriophages and mammalian viruses may limit direct quantitative extrapolation. In this context, the use of a batch scale‐down model remains essential for assessing how variables such as buffer composition, ionic strength, and product titer influence viral inactivation kinetics and product quality.^45^, ^46^

The use of Phi6 allows for the rapid accumulation of process know‐how and technical data, directly addressing the regulatory hesitancy associated with continuous processing. Establishing this robust technical basis with Phi6 streamlines the eventual confirmatory studies required by ICH Q5A(R2), as the system's performance and inactivation boundaries are already well‐characterized. Consequently, Phi6 can serve as a strategic tool to de‐risk the transition to intensified downstream manufacturing while ensuring that automation and continuous operation do not compromise product quality.

## 5 CONCLUSION

This study demonstrates that membrane adsorbers serve as an effective plug‐and‐play solution for intensified biomanufacturing, significantly increasing profitability by reducing capital and operational costs. A primary driver of this efficiency is the combination of increased productivity and DOE‐optimized buffer consumption, which maintains robust product quality and high step yields while eliminating the underutilization common in traditional resin platforms. The high throughput and short cycle times of this capture mode facilitate the immediate transition to integrated continuously operated low pH virus inactivation. This continuous setup achieved phage inactivation levels comparable to both traditional batch modes and reported literature values for mammalian model viruses. While these results establish a robust technical foundation for intensified downstream processing, further validation using this system and the established process control strategy with ICH‐recommended mammalian model viruses remains the necessary next step.

## AUTHOR CONTRIBUTIONS

**Mario Grünberg:** Conceptualization, Methodology, Data curation, Supervision, Formal analysis, Visualization, Writing—original draft, Investigation. **Lisa Lipski:** Conceptualization, Methodology, Data curation, Formal analysis, Investigation, Writing—original draft. **Gabriel Fisicaro:** Conceptualization, Methodology, Project administration, Writing—review and editing. **Thomas Duvignau:** Conceptualization, Methodology, Project administration, Writing—review and editing. **Karolina Meyer‐Heinrichs:** Methodology, Data curation, Writing—review and editing. **Diana Carmen Mocsy:** Methodology, Data curation, Formal analysis, Writing—review and editing. **Thomas‐Josef Filz:** Software, Data curation, Methodology, Writing—review and editing. **Bastian Quaas:** Methodology, Data curation, Investigation, Visualization, Writing—review and editing. **Alexandra Stützer:** Conceptualization, Methodology, Software, Writing—original draft, Resources, Supervision.

## FUNDING INFORMATION

This work received no specific funding from public agencies, or not‐for‐profit organizations and was not supported by any external funding sources.

## CONFLICT OF INTEREST STATEMENT

M. Grünberg, L. Lipski, K. Meyer‐Heinrichs, D. Mocsy, T.‐J. Filz, B. Quaas and A. Stützer were employees of Sartorius, Goettingen, Germany at the time the work was performed. G. Fisicaro and T. Duvignau were employees of LFB Biomanufacturing, Ales, France at the time the work was performed.

## Supporting information


**Data S1:** Supplementary Information

## Data Availability

The data that support the findings of this study are available from the corresponding author upon reasonable request.
