# Supplementary material for: Design of experiments‐guided membrane capture facilitates integration of continuous virus inactivation using Phi6 as a surrogate virus in an intensified downstream process
Source: Biotechnol Prog. 2026 Mar 25;42(3):e88504. doi: 10.1002/btpr.88504 (PMC13266959; doi:10.1002/btpr.88504)
Supplement: Supplementary file 1 — Data S1: Supplementary Information [file BTPR-42-e88504-s001.docx]

**SUPPORTING INFORMATION**

**Supplementary Information (Materials and Methods)**

**Bacteriophages Phi6 production, spike and quantification**

For bacterial cultivation, the freeze-dried bacterial pellet was dissolved in 5 ml of tryptone soy broth (TSB), followed by preparation of a three-eyelet smear from the bacterial suspension, which was incubated at + 25°C for 44 to 48 hours. Subsequently, a colony of *P. syringae* was inoculated into 20 ml of TSB and incubated overnight in a shaker at 25°C. Once the OD_600_ exceeded 1.0, aliquots were prepared with 400 μl of bacterial suspension and 100 μl of 86% glycerol, then flash frozen in cryo-tubes using liquid nitrogen. The aliquots are stored at - 80°C.

For phage cultivation, the frozen bacterial stock was thawed to room temperature, and a three-eyelet smear was prepared from the bacterial suspension, incubated at 25°C for 44 to 48 hours. Subsequently, a colony of *P. syringae* was inoculated into 20 ml of tryptone soy broth (TSB) and incubated overnight in a shaker at 25°C. Following this, the overnight culture was transferred into TSB and incubated in a shaker for 24 to 48 hours at 25°C, with continuous monitoring of OD_600_ until it reached a value between 0.6 and 1.0. Bacteriophages were then added to the bacterial suspension and incubated at 25°C for 24 to 48 hours in a shaker. Following incubation, bacteriophages were collected by centrifugation at 4,000 rpm for at least 30 minutes. The supernatant containing the bacteriophages was filtered through 0.2 μm and 0.1 μm filters. Afterwards 9 ml Phi6 suspension were mixed with 1 ml 86 % glycerol, shock-frozen in liquid nitrogen, and stored in aliquots at - 80°C. An aliquot was utilized to determine the phage concentration [pfu] by conducting a plaque assay.

Samples containing Phi6 were serially diluted in tryptic soy broth and incubated with *P. syringae* overnight cultures. After a 10-minute incubation at room temperature, top agar was added and the mixture was plated. Two controls were included to verify media sterility and absence of phage contamination. Plates were incubated at room temperature for 42 to 48 hours. Plaques were counted and titers calculated based on dilution and sample volume.

**Surrogate continuous low pH virus inactivation**

All experiments were conducted in a climate-controlled chamber at 18 °C and 50% relative humidity. Prior to testing titration curves for acidification were determined offline and uploaded to the system software.

To evaluate the performance of the continuous virus inactivation process, experiments were conducted at two residence times: 35 minutes and 70 minutes, corresponding to flow rates of 12 L/h and 6 L/h, respectively. Phage solution was introduced after 50 minutes (12 L/h) or 85 minutes (6 L/h) of pre-flow of eluate at pH 3.6 to ensure proper saturation of the incubator. The sampling ports corresponded to different sampling time points depending on the set flow rate (7, 14, 21, 28, 35 min for 12 L/h; 0, 14, 28, 42, 56, 70 min for 6 L/h). For both setups, the phage stock solution (9.9×10⁹ pfu/mL) was introduced via the peristaltic pump to reach the target inline concentration of 5×10⁷ pfu/mL. Back-to-front sampling was performed with samples immediately neutralized using 1 M Tris-HCl pH 9 to maintain pH between 6.5 and 7.5. Duplicate samples were collected for each time point for HPLC analysis and phage titer determination. A negative control was conducted at pH 5.0, and the sample was taken at the sampling port located upstream of the incubator for both flow rates to determine the initial phage titer. This measurement was used as the initial titer to calculate LRV.

**Quantification of mAb titer and Monomer**

The mobile phase SEC-HPLC consisted of 50 mM Na_2_HPO_4_, 100 mM Na_2_SO_4_ pH 6.4. The flow rate was maintained at 0.3 mL/min, with sample injection volumes of 5 µl. Harvested cell culture fluid (Protein A feed) samples were analyzed for 40 minutes (to prevent carry-over of sample components in subsequent sample), while Protein A eluate samples were analyzed for 20 minutes. Chromatographic detection was performed at 280 nm using a variable wavelength detector (VF-D40-A, Thermo Fisher Scientific, USA). For mAb quantification, a Protein A resin eluate fraction was used for calibration, quantified using an extinction coefficient of A_280_(0.1%) = 1.65 AU. The eluate fraction was diluted to concentrations of 10, 7.5, 5, 3.75, 2.5, 1, 0.5, 0.25, 0.125, and 0 g/L in the mobile phase to establish a linear calibration curve
